# Comparison of Nutri-Score and Health Star Rating Nutrient Profiling Models Using Large Branded Foods Composition Database and Sales Data

**DOI:** 10.3390/ijerph20053980

**Published:** 2023-02-23

**Authors:** Edvina Hafner, Igor Pravst

**Affiliations:** 1 Nutrition Institute, Tržaška Cesta 40, SI-1000 Ljubljana, Slovenia; 2 Biotechnical Faculty, University of Ljubljana, Jamnikarjeva 101, SI-1000 Ljubljana, Slovenia; 3 VIST—Faculty of Applied Sciences, Gerbičeva Cesta 51A, SI-1000 Ljubljana, Slovenia

**Keywords:** labelling, front-of-pack, branded foods database, sale-weighting, Nutri-Score, Health Star Rating

## Abstract

Front-of-package nutrition labelling (FOPNL) is known as an effective tool that can encourage healthier food choices and food reformulation. A very interesting type of FOPNL is grading schemes. Our objective was to compare two market-implemented grading schemes—European Nutri-Score (NS) and Australian Health Star Rating (HSR), using large Slovenian branded foods database. NS and HSR were used for profiling 17,226 pre-packed foods and drinks, available in Slovenian food supply dataset (2020). Alignment between models was evaluated with agreement (% of agreement and Cohen’s Kappa) and correlation (Spearman rho). The 12-month nationwide sales-data were used for sale-weighing, to address market-share differences. Study results indicated that both models have good discriminatory ability between products based on their nutritional composition. NS and HSR ranked 22% and 33% of Slovenian food supply as healthy, respectively. Agreement between NS and HSR was strong (70%, κ = 0.62) with a very strong correlation (rho = 0.87). Observed profiling models were most aligned within food categories Beverages and Bread and bakery products, while less aligned for Dairy and imitates and Edible oils and emulsions. Notable disagreements were particularly observed in subcategories of Cheese and processed cheeses (8%, κ = 0.01, rho = 0.38) and Cooking oils (27%, κ = 0.11, rho = 0.40). Further analysis showed that the main differences in Cooking oils were due to olive oil and walnut oil, which are favoured by NS and grapeseed, flaxseed and sunflower oil that are favoured by HSR. For Cheeses and cheese products, we observed that HSR graded products across the whole scale, with majority (63%) being classified as healthy (≥3.5 *), while NS mostly graded lower scores. Sale-weighting analyses showed that offer in the food supply does not always reflect the sales. Sale-weighting increased overall agreement between profiles from 70% to 81%, with notable differences between food categories. In conclusion, NS and HSR were shown as highly compliant FOPNLs with few divergences in some subcategories. Even these models do not always grade products equally high, very similar ranking trends were observed. However, the observed differences highlight the challenges of FOPNL ranking schemes, which are tailored to address somewhat different public health priorities in different countries. International harmonization can support further development of grading type nutrient profiling models for the use in FOPNL, and make those acceptable for more stake-holders, which will be crucial for their successful regulatory implementation.

## 1. Introduction

Strong evidences show that dietary factors are linked to the development of non-communicable chronic diseases [1,2]. Therefore, improving people’s diets is an important public health goal. In high-income countries, consumption of ultra-processed prepacked foods and drinks is increasing [3,4,5], while the nutritional quality of such products is often questionable [6].

Two key approaches for improving people’s diet are encouraging them to choose healthier foods (e.g., education, promotion) and the implementation of policies, which stimulate the development of foods with favourable nutritional composition (e.g., food reformulation policies, taxation, …) [7]. One way to address these approaches is through food labelling, representing the link between the consumer and the manufacturer. In recent years, different authorities emphasize the importance of front-of-package nutrition labelling (FOPNL) [8,9]. FOPNL should enable the consumer to distinguish quickly and clearly between foods according to their nutritional composition [10]. Such labelling can empower consumers to recognize differences between similar pre-packed foods and encourages manufacturers to reformulate their products [11], i.e., lower content of saturated fat, sugar, and sodium.

However, not all FOPNLs are equally effective. FOPNL schemes vary based on their graphic representation and parameters, which are included in the nutrient profiling algorithm. In recent times, emphasis has been placed on interpretive FOPNL schemes, which can subconsciously direct consumers towards healthier options (e.g., use of traffic light colours, rating, and heart symbol) [12,13]. Such labelling could support all consumers (regardless of their background nutritional knowledge) with making not only informed but also healthier food choices. The effectiveness of such schemes was supported by recent studies [14,15].

The European Commission (EC) is currently discussing the implementation of mandatory harmonized FOPNL across the EU region [16]. One type of interpretive FOPNL are overall grading schemes [10], which became interesting for public health policy makers as they provide simplified information (easier for interpretation), can be used on all products, and they avoid dichotomous thinking that foods can be either healthy or unhealthy [17]. Only two such schemes are currently used on the market: Nutri-Score (NS) in Europe, and Health Star Rating (HSR) in Australia. NS is an overall grading scheme developed in France, which grades foods on a scale from A-dark green (healthiest) to E-dark orange (least healthy) [18]. NS was developed with the considerations of the market; it is one of the most scientifically supported voluntarily FOPNLs, successfully implemented in several European countries, and therefore one of the candidates for the introduction of harmonized FOPNL in Europe. On the other hand, HSR is a voluntary scheme developed in Australia by the government in collaboration with the food industry and consumers groups. It grades foods in half-star increments from 0.5 * (least healthy) to 5 * (most healthy) and is commonly used in scientific research to evaluate the healthiness of foods and drinks [19]. Both schemes were built on the Ofcom Nutrient Profiling System, which was established to limit the marketing of unhealthy foods to children in the United Kingdom [20]. Even though both schemes have a very similar core system, adaptations made in the development process affected the way how food products are presented to consumers. For example, NS is 5-colour graded FOPNL, while HSR in the monochrome system with 10 possible star grades. Unlike NS, which only included minor changes to Ofcom profiling, HSR extended score scales for most of the attributes included in the profiling. The main differences between NS and HSR are described in Table 1.

Even though rating schemes can be easier for interpretation, some researchers are noting limited ability of FOPNLs for long-term changes into healthier dietary patterns, and emphasize the importance of consumer education. Some challenges were also highlighted for NS and HSR [21,22]. However, to our knowledge, no study so far did a comparison of these two rating systems across whole food supply. Such comparison would give us valuable data, highlighting the main challenges of grading schemes and opportunities for their improvement. Considering that FOPNLs have been particularly emphasized in the last few years, there is a lack of real-life data. Furthermore, the use of individual foods market-shares was shown to be useful for monitoring critical nutrients, but such an approach can be also used to examine what kind of products consumers are exposed to.

The aim of this study was to compare two grading FOPNL schemes—NS and HSR, which both aim to promote healthier foods and beverages to the consumer. We wanted to evaluate the alignment between these two systems and identify the main differences and related challenges, based on nationally representative branded food composition database. Market-share differences were addressed with the use of a sale-weighting approach, with consideration of 12-month sales data.

## 2. Materials and Methods

### 2.1. Data Sources

The study included cross-sectional data on available pre-packed foods from the Slovenian food supply (Slovenian branded foods dataset, 2020). The data source was the Composition and Labelling Information System (CLAS, Nutrition Institute, Ljubljana, Slovenia). The detailed methodology on data collection for CLAS is described elsewhere [23]. Briefly, CLAS includes the photo collection of labels of pre-packed foods from major retailers that cover the majority of the food market share in Slovenia. It includes all products with unique barcodes (most commonly in the form of the Global Trade Item Number—GTIN) that were available at the time of sampling. The 2020 dataset included 28.028 pre-packed foods from 7 stores all located in Ljubljana, Slovenia: two mega-markets (Interspar, Mercator Center), two supermarkets (Tuš, Spar), and three discount markets (Eurospin, Hofer, Lidl). Information on nutritional composition and ingredients were extracted from the photos.

Yearly product specific sales data for 2020 were obtained from retailers, which represent over 50% of the national market share. With the help of GTIN barcodes, sales data were matched with the CLAS database. Using package size, we were able to calculate the quantity (kg/L) of product sold in a year, which was used for sale-weighting.

### 2.2. Product Categorization and Exclusion Process

Products in CLAS were categorized according to the international food categorization developed by the Global Food Monitoring group, to enable the monitoring of pre-packed foods and beverages [24]. The categorization was slightly modified according to European market specifics and study methodology. Product exclusion consisted of four steps. At first, we excluded foods from categories that are not covered by NS or HSR (N = 9.250). Then, we excluded products with missing data, which is a part of mandatory nutritional declaration (N = 881) and products that needed preparation, which would require the addition of other ingredients (N = 546). To avoid possible errors in the database, we also excluded products where calculated energy content (based on the content of nutrients) was differentiated from the labelled energy content for over ±20% (N = 125). These calculations were conducted with a protocol provided in the Regulation (EU) No. 1169/2011 [25]. After the exclusion process, we ended up with 17,226 products from 12 main food categories and 53 subcategories.

### 2.3. Dealing with Missing Data

Since nutrient profiling requires some information, which is not part of mandatory food labelling, our dataset was supplemented with missing data for fibre, content of fruit, vegetables, nuts, and legumes (% FVNL), and calcium content.

The determination of missing dietary fibre and % FVNL content was performed as described elsewhere [26]. For dietary fibre, we defined food categories where fibre could be above 0.9 g/100 g, where the dietary fibre content becomes relevant for NS and HSR. The missing fibre content for relevant categories was then calculated for each product based on its energy value, content of other nutrients and their energy factors, using a previously described method [25]. fibre content was calculated for 2814 (16%) products. The % FVNL was assessed using ingredient lists, and sometimes with consideration of the legislation [27,28]. We separately determined the content for specific oils important to NS (olive, walnut, and rapeseed oil), concentrated, dried and fresh FVNL, which was then converted into the total % FVNL by using the method described in the profiling protocol [29,30].

The calcium content was needed for the calculation of HSR for dairy products. When possible, the calcium content was extracted from the label; otherwise, it was assessed based on similar products (e.g., same type of cheese) available in national food composition database Open Platform for Clinical Nutrition (OPKP) [31]. Alternatively, the Fineli [32] database was used. For dairy imitates, the calcium content was only taken into account if the product had declared calcium content on the label.

Profiling exceptions were done with the use of food name and ingredient lists (i.e., plain water (for both models), water-based ice confections, minimally processed fruits and vegetables, and unsweetened flavoured waters (for HSR)).

### 2.4. Calculation of the NS Grade

Calculations of NS grades were performed in the alignment with Scientific and Technical instructions of French Public Health Agency [29]. At first, we assigned products to one of the four NS profiling categories (General, Beverages, Added fats, and Cheeses). Then, based on nutritional composition per 100 g or 100 mL, we assigned positive points (0–40) to negative attributes and negative points (0–15) to positive attributes. Negative attributes were energy (kJ), total sugars (g), saturated fatty acids (g), and sodium, while positive attributes include fibre (g), proteins (g), and % FVNL. The sum of positive and negative attributes gave the products a final score. Based on the final score, the product was graded from A (healthiest) to E (least healthy).

### 2.5. Calculation of the HSR Grade

Calculations of the HSR grades were performed according to the criteria provided by the Front-of-Pack Labelling Secretariat of the Australian Government [30]. Products were assigned to one of six HSR profiling categories: 1: Non-dairy beverages, 2: Foods, 3: Oils and spreads, 1D: Dairy beverages, 2D: Dairy foods, and 3D: Cheeses. Similar to NS, points were assigned to the same positive and negative attributes, but the amount of maximum points varied from 10 to 30 for each negative attribute and 8 to 15 for each positive attribute (depending on the profiling category). The sum of all negative and all positive points resulted in the product final score, based on which product was graded to one of ten HSR grades: from 5 stars (*) (healthiest) to 0.5 stars (*) (least healthy).

### 2.6. Data Analyses

Statistical analyses were performed using Microsoft Excel 2019 (Microsoft, Redmond, WA, USA) and R Studio (R Core Team, Vienna, Austria).

HSR has ten possible grades, while NS only has five. For easier comparison, we combined HSR grades into five, which were comparable to NS (HSR grades 5 * and 4.5 * were joined and compared to NS grade A, 4 * and 3.5 * with B, and so on). In line with previous studies, the cut-off value for healthier products was set at 3.5 * or more for HSR, and A/B for NS [33,34,35].

First, we assessed the distribution of different NS and HSR grades and overall healthiness of the Slovenian food supply. We compared both models based on available foods and based on sale-weighted distribution, and assessed their ability to discriminate products within a specific food (sub)category. The ability to discriminate between products was defined as good when there were at least three grades within a specific (sub)category [36].

The alignment between NS and HSR was assessed with the calculation of agreement (% of agreement and Cohen’s Kappa) and correlation (Spearman rho). At first, we calculated the % of agreement. If the model had sufficient discriminatory ability, we continued assessing agreement with Cohen’s Kappa. We have considered that models may not grade products equally high, but the way the products are ranked from the best to the worst can be similar. For this purpose, we also calculated the correlation with Spearman rho. Cut-off ranges for agreement and correlation were as follows (multiplied by 100 % for % of agreement): 0–0.20, negligible; 0.21–0.40, weak; 0.41–0.60, moderate; 0.61–0.80, strong; 0.81–1, very strong [33,37]. We then identified food categories that had the worst agreement and correlation by using all three approaches. These categories were subject to further categorization [38,39] and were then presented with descriptive statistics.

## 3. Results

Our final sample consisted of 17,226 pre-packed products. The largest share of the sample represented Dairy and imitates (18.6%), followed by Bread and bakery products (13.4%), Confectionery (13.3%), Meat and meat products (11.0%), Beverages (10.6%), Fruits and vegetables (8.4%), Sauces and spreads (7.0%), Convenience foods (4.4%), Snack foods (3.5%), Edible oils and emulsions (3.4%), Fish and fish products (3.3%), and Cereal and cereal products (3.2%) (Table 2).

### 3.1. Discriminatory Ability and Distribution of Grades across Food Supply

**Figure 1:**
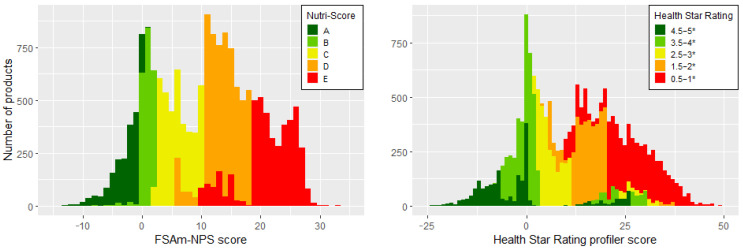
Overall distribution of profiling scores according to Nutri-Score (FSAm-NPS (Food Standard Agency- Nutrient Profiling System)) and Health Star Rating algorithm in conjunction with their final grade. Colours represent different Nutri-Score and Health Star Rating grades across 2020 Slovenian food supply (n = 17,226). * refers to stars of Health Star Rating grades.

The overall distribution of NS and HSR grades of products in the Slovenian food supply is displayed in Figure 1. NS graded 10% of products A, 12% B, 20% C, 32% D, and 25% E. HSR grades were slightly in favour of healthier foods: 12% of products were graded 4.5–5 *; 21%, 3.5–4 *; 16%, 2.5–3 *; 22%, 1.5–2 *; and 29%, 0.5–1 *. It should be noted that, in HSR, products with a higher score (>20), which are otherwise associated with negative grades, can get a final grade of 4.5–5 *. This reflects the modifications of HSR for dairy and oils and spreads. Using criteria A/B for NS and 3.5 * for HSR [33,34,35], the NS model ranked 22% of products in the Slovenian food supply as “healthy”, while HSR ranked 33% of the products as healthy. The discriminating ability (3 or more different grades within a category) was good in all the main categories for both NS and HSR and in 91% of subcategories for NS and 94% for HSR. The discriminating ability was low in some smaller homogenous subcategories such as Water, Frozen fruit, and Frozen vegetables, for both NS and HSR, and for NS also in categories of Butter and Animal fat products.

**Figure 2:**
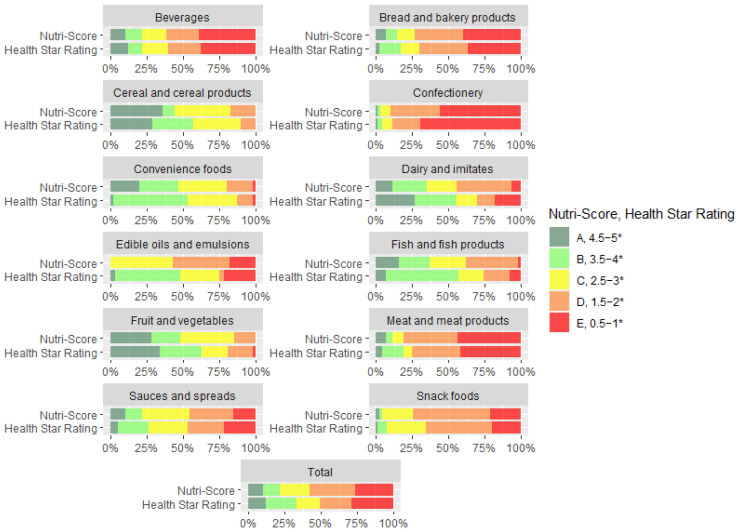
Distribution of Nutri-Score and Health Star Rating grades across main categories. Colours represent different Nutri-Score and Health Star Rating grades. * refers to stars of Health Star Rating grades.

The distribution of NS and HSR across main categories is displayed in Figure 2. In the main categories, NS was stricter in grading Convenience foods, Dairy and imitates, Fish and fish products, Meat and meat products, Edible oils and emulsions, and Snack foods. However, HSR was stricter than NS for the highest rated products (4.5–5 * or A) in categories of Cereal and cereal products, Convenience foods, Fish and fish products, Meat and meat products, and Sauces and spreads. HSR was also stricter for Confectionery, rating 69% of the available foods with lowest grades 0.5–1 *, while NS rated 56% with a grade E. The most notable difference in grading was observed in categories of Edible oils and emulsion and Dairy and imitates. For Edible oils and emulsions, HSR graded almost half (48%) of the products as “healthy” while for NS, none of these products were assessed as healthy (highest grade was C). Similar results were observed for Dairy and imitates, where HSR assessed 56% of products “healthy”, while NS only 35%. There were no notable differences in the rating of Beverages.

Differences become clearer, when results of modelling are compared for food subcategories (Supplementary Material S1), especially in Dairy and imitates. In Cottage cheese, HSR graded 73% of products with 4.5–5 *, while NS rated less than half of the products (47%) with A. In Cheese and processed cheese, NS in vast majority (82%) rated products D, 12% C, and a few products B (1%) and E (5%). HSR distributed such products across all grades: 35% with grades 4.5–5 *; 28%, 3.5–4 *; 8%, 2.5–3 *; 6%, 1.5–2 *; and 22%, 0.5–1 *. NS showed good discriminating ability in the category of Plain yogurts, where full fat yogurts got a B (38%), and skimmed yogurts got an A (50%). HSR rated 83% of products 4.5–5 *, with full fat yogurts mostly getting a 4.5 * and skimmed yogurts a 5 *. In the category of Flavoured yogurts, HSR rated products higher than NS, with 19%, 4.5–5 *; 54%, 3.5–4 *; 24%, 2.5–3 *; and 2%, 1.5–2 *. NS rated Flavoured yogurts mostly B (46%) or C (45%), and some products with A (8%) and D (1%).

In the main category of Convenience foods, NS was slightly stricter from HSR in rating Pizza; however, HSR was stricter when it comes to the highest grades (4.5–5 * or A) for Ready meals, Pre-prepared salads and sandwiches, and Side dishes.

For the subcategory of Cooking oils, NS was stricter and graded products with C (51%), D (40%), and E (9%), while HSR distributed such products across all grades: 2% with grades 4.5–5 *; 54%, 3.5–4 *; 33%, 2.5–3 *; 1%, 1.5–2 *; and 10%, 0.5–1 *. Another interesting category where we observed notable differences were Breakfast cereals, where HSR assessed 51% of products as “healthy”, while for NS, this was 37%. In the category of Beverages, the results of profiling were most aligned, with some smaller differences. In the subcategory of Nectars, HSR graded some products better than NS due to the lower threshold for % FVNL. We also observed that HSR made notable differences in grading unsweetened flavoured waters (4.5 *), 100% fruit juices with lower sugar content (<7 g per 100 mL) (4 *), and drinks with non-caloric sweeteners (3.5 *), while for NS, such drinks were mostly graded B.

### 3.2. Agreement and Correlation between Nutri-Score and Health Star Rating

**Figure 3:**
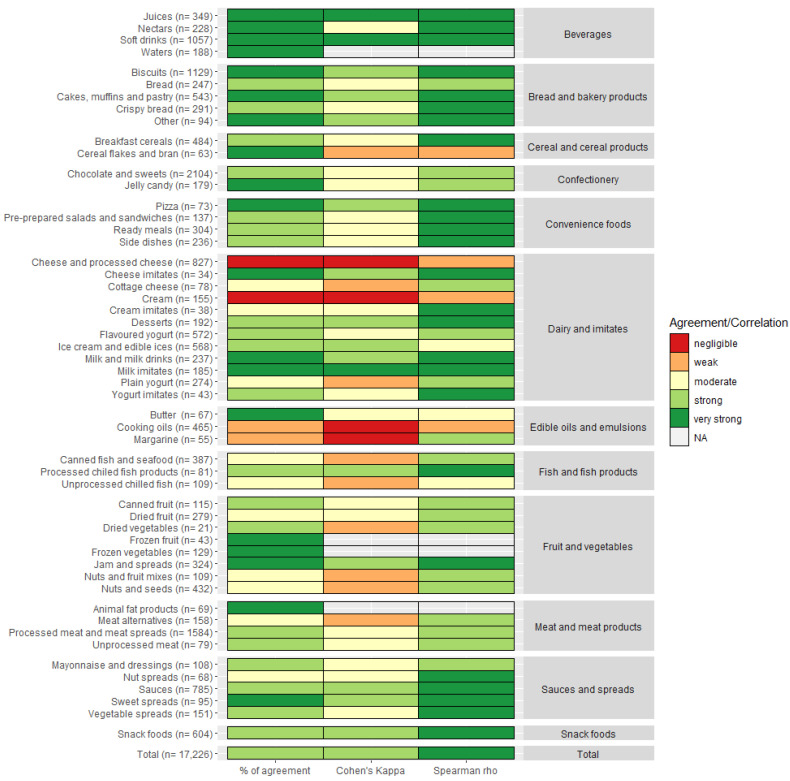
Agreement (% of agreement and Cohen’s Kappa) and correlation (Spearman rho) between Nutri-Score and Health Star Rating for subcategories. The main categories of the analysed subcategories are presented on the right. Cut offs ranges for agreement and correlations were as follows (multiplied by 100% for % of agreement): 0–0.20 negligible (red); 0.21–0.40 weak (orange); 0.41–0.60 moderate (yellow); 0.61–0.80 strong (light green); 0.81–1 very strong (dark green); NA—not applicable.

The evaluation of alignment based on agreement and correlation between NS and HSR for subcategories is displayed in Figure 3, with exact values available in Supplementary Material S1. Overall, we determined strong agreement (70% and κ = 0.62) and very strong correlation (rho = 0.87) between both models. The percentage of agreement was perfect (100%) for Waters, Frozen fruit, and Frozen vegetables, where both models rated all products with the highest possible grade. The percentage of agreement was also very strong (81–100%) for 16 other subcategories, especially in the main categories of Beverages and Bread and bakery products and in other more homogenous subcategories such as Cereal flakes and bran (94%), Butter (93%), Jelly candy (86%), and Milk and milk drinks (82%). We observed that 13 subcategories had moderate to strong % of agreement and kappa, but very strong correlation, which indicates that models do not always agree on the grade, but still rank products similarly. This includes subcategories such as Breakfast cereals (rho = 0.83), Crispy bread (rho = 0.89), Cream imitates (rho = 0.85), Desserts (rho = 0.88), Yogurt imitates (rho = 0.90), Snack foods (rho = 0.82) and most subcategories in the main categories of Convenience foods and Sauces and spreads. We identified that NS and HSR have either very strong agreement or correlation in 32 (60%) subcategories. A slightly lower agreement but still strong correlation was found in most subcategories of Fruit and vegetables and Meat and meat products. For Canned (78%, κ = 0.53, rho = 0.71) and Dried fruit (59%, κ = 0.43, rho = 0.79), HSR rated products were slightly stricter due to a higher penalization of sugars. For Nuts and fruit mixes (48%, κ = 0.23, rho = 0.65) and Nuts and seeds (50%, κ = 0.31, rho = 0.78), HSR graded products higher due to a higher contribution of positive components (fibre, % FVNL and protein), in comparison to NS. HSR was stricter in grading Unprocessed meat and Meat alternatives, and NS was stricter in grading Processed meat and meat products. Five categories with the lowest alignment between models were Cheese and processed cheese (8%, κ = 0.01, rho = 0.38), Cream (14%, κ = 0.03, rho = 0.26), Cooking oils (27%, κ = 0.11, rho = 0.40), Margarine (27%, κ = 0.13, rho = 0.72), and Unprocessed chilled fish (54%, κ = 0.30, rho = 0.55). We observed that differences between models for Unprocessed chilled fish were due to extended HSR scale for protein. Most such products got maximum points for protein with NS, while for HSR protein, points differ substantially between products, making HSR a stricter profiling model for the highest grades (4.5–5 *). For Margarine, NS was much stricter (highest grade C) but the overall ranking/correlation was strong. The subcategory of Cream was a small and homogenous group, where most products were graded the same. NS graded most of these products (86%) D and HSR (81%) 0.5–1 *, which resulted in poor agreement. Two subcategories with the lowest alignment (and with enough diverse products for further analysis) were Cheese and cheese products and Cooking oils.

### 3.3. Main Differences in Grading with NS and HSR

**Figure 4:**
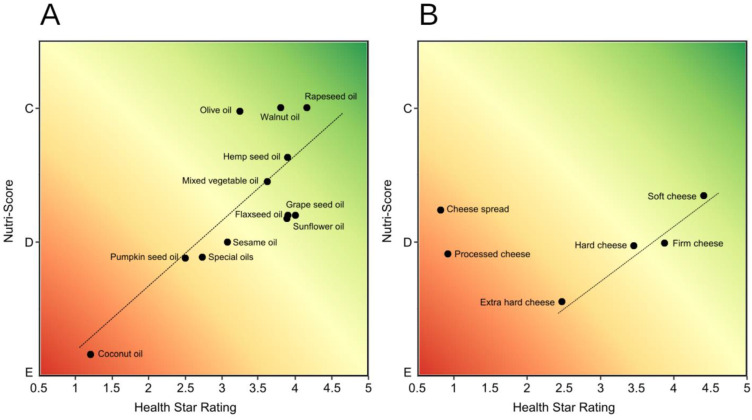
Average NS and HSR for different types of Cooking oils (**A**) and Cheeses (**B**). Special oils include less common oils (n < 5), such as black cumin oil, apricot oil, and argan oil.

Categories of Cheese and processed cheese and Cooking oils, where major differences were observed for profiling with NS and HSR, were subject to further descriptive analysis (Figure 4). After dividing products according to the type of oil/cheese, we could determine the type of products on which the models agree and disagree. In Cooking oils, the models were in alignment especially for coconut oil, pumpkin seed oil, mixed vegetable oil, and hemp seed oil. Smaller differences in grading were observed for special oils, sesame oils, and rapeseed oils, but the overall ranking stayed the same. The main differences were observed for olive oil and walnut oil, which are favoured by NS (mostly graded C) and grapeseed, flaxseed, and sunflower oil, which are favoured by HSR (mostly graded 3.5–4 *). For Cheeses and cheese products, we observed that HSR grades products across the whole scale, with the majority (63%) being ranked as healthy (≥3.5 *). On average, products that were defined as healthy were soft, firm, and hard cheeses, while less healthy were extra hard cheeses, processed cheese, and cheese spreads. On the other hand, NS graded most of the products with lower grade D (82%). The highest average grade had soft cheeses with some graded C (n = 69), and the lowest grade for extra hard cheeses. The most notable difference was observed for processed cheese and cheese spreads, with typical HSR grades 0.5–1 *, while NS graded them similar to other cheeses (mostly D).

### 3.4. Accounting for Market Share-Differences

To examine agreement between both profiling models with consideration of market-share differences of individual foods, we used 12-month national sales data for the year 2020. Sales data were available for 62% (n = 10,724) of the products in our dataset, which were used for the assessment. Our results highlighted (Supplementary Material S1) that the food availability does not necessarily reflect the sales. This is not surprising in the food supply, where some market-leading foods can have magnitudes of higher sales in comparison to some niche and specialty products. The sale-weighing approach enabled us to account for these differences, prioritizing market-leading products. After sale-weighting, categories of Beverages, Bread and bakery products, Convenience foods, Dairy and imitates and Fruit and vegetables showed higher proportion of healthy products (NS A or B or HSR ≥ 3.5 *) in comparison to the product availability, while the contrary was observed for Confectionery, Cereal and cereal products, Fish and fish products, Meat and meat products, Snack foods and Sauces and spreads. The overall % of agreement between NS and HSR increased after sale-weighting from 70% to 81%. The percentage of agreement between models was increased after sale-weighting for 8 out of 12 main categories and for 26 out of 53 subcategories. The subcategories where agreement notably decreased were Side dishes (from 67% to 32%), Plain yogurt (from 53% to 33%), Desserts (from 72% to 49%), and Cooking oils (from 27% to 6%).

## 4. Discussion

The current study showed that both the NS and HSR profiling models have good discriminatory abilities and are in very good alignment for most prepacked foods in the Slovenian food supply. However, there notable differences were observed in some subcategories, particularly in Cheese and processed cheese and Cooking oils.

Differences in some subcategories affected the overall grading of the food supply. Using both models, we saw that NS was the stricter model, grading 11% less food supply as “healthy” in comparison to HSR. The evaluation of the healthiness of the products in the food supply is investigated in many studies, and is also an indicator of the food environment and efficiency of public health policies and interventions [40]. The diversity of nutrient profiling models used in research, and lack of a globally harmonized profiling model is limiting comparisons between countries and between different studies, because models were developed with different priorities [33]. Even for similar nutrient profiling models, such as NS and HSR, such challenges were noticeable, which emphasizes how the very choice of the profiling method affects the result. This should be considered when discussing the healthiness of the products in the food supply.

Overall, NS and HSR showed strong agreement and a very strong correlation. Similarities were observed, especially in categories of Beverages. In the year 2020, the HSR model was adapted for beverages, based on the NS algorithm, which explains very good alignment in this category. Alignment was also very good in categories of Bread and bakery products, Convenience foods, Sauces and spreads, Snack foods and for the majority of Dairy and imitates. For most categories, we observed that even though the grades were not always equally high, within the category ranking was comparable for both the models. Differences between NS and HSR mostly resulted from HSR extended scoring scales and adjustment of the profiling for specific food groups. The effect of extended scoring scales was particularly noticeable in products with high content of specific nutrients. For example, Nuts and seeds mostly have high protein and fibre content. Therefore, most products get maximum points for these attributes when profiling with NS. Using HSR, extended scales indicate such products can get more positive points, resulting in a greater impact of these attributes on the grade and overall better grading for this category using HSR (85% assigned as healthy). The same applies to negative points. Greater penalization of sugars and saturated fatty acids using HSR resulted in slightly stricter profiling, notable in Confectionery. Even though extended scales can be an advantage and address some of the issues that have already been highlighted for NS, caution is required when it comes to products, for which are very high in energy or some nutrient. Because of high fibre and protein, Nuts and seeds mostly get a HSR grade of 3.5 * or more (healthier products), even though some products have added salt or sugar. An extended scale for protein can also result in relatively high grades of Processed meat and meat products, a controversial category, for which limited consumption is recommended [41]. In Breakfast cereals, we observed that the inclusion of positive components, such as dietary fibre and protein, can “cancel out” the high amount of sugar to some extent. HSR, therefore, rated 51% Breakfast cereals as healthy, while NS only 37%. While the higher number of star ratings allows HSR to still distinguish between products within a category, consumers might perceive higher (“healthier”) ratings differently. Pelly et al. (2020) explored consumers’ perception of HSR. They reported that even though consumers find HSR useful, some scepticism was highlighted towards the profiling algorithm, when it came to discretionary foods, which they felt were often rated too high [42]. Consumers’ trust and support is a key aspect that can determine efficiency and even survival of the specific FOPNL. Such an example is the Choices symbol, which was implemented in the Netherland and well known by more than 90% of Dutch consumers. However, changes in the profiling algorithm, and particularly the use of modified symbol on discretionary foods, were not well understood among consumers and criticized extensively by consumer organizations. Loss of credibility with consumers and the government resulted in phasing out the logo in the Netherland [43]. This indicated that, in addition to discriminatory ability, it is also important to consider that grading of foods is aligned with the consumer’s logic.

Categories where grading is often exposed as questionable are dairy products and edible oils [21,44]. Our study also highlighted major differences in the nutrient profiling of Cheese and processed cheese and Cooking oils between NS and HSR. Differences in these categories were also found in a study, when a comparison was performed with the WHO Europe nutrient profiling system [26]. This reflects adjustments in the nutrient profiling, which were not established to the same extent in different models. For Cooking oils, NS kept the threshold for the final score based on the profiling of general foods, while adding positive points in the form of % FVNL for olive, walnut and rapeseed oils, which had the biggest impact on the overall grading. Meanwhile, HSR changed thresholds for the final grade, but grading is still based only on the nutritional composition, without consideration of the content of specific oil types. The changed thresholds resulted in HSR being a less strict model and the grading of oils being distributed over all grades, while the NS graded oils with C or lower, giving the preferences to olive, walnut and rapeseed oil (grade C) ahead of other oils (grades D and E). NS adjusted the system based on the French Agency for Food, Environmental and Occupational Health & Safety (Agence nationale de sécurité sanitaire de l’alimentation, de l’environnement et du travail—ANSES) nutritional recommendations, which highlighted the advantages of mentioned oils, such as higher content of omega 3 fats and polyphenols [45]. Many stakeholders suggested similar modifications for the HSR system. After a five-year review, the HSR remained the same with an argument that the HSR calculator does not consider components such as polyphenols, omega-3 fats, and vitamins, and it would be inappropriate to highlight these constituents only for the category of oils [46]. However, HSR does include calcium as an important component for profiling dairy products, but not in other categories. A customized profiling method for these products led to HSR having better discriminatory ability especially for Cheese and processed cheese, distributing products across all grades, while NS mostly graded such products with less favourable grade D. The inclusion of some micronutrients (i.e., potassium) and other components in nutrient profiling has been also encouraged recently by the European Food Safety Authority (EFSA) [47], but such modifications are challenging, when it comes to constituents that are not part of mandatory food labelling, and where estimations would not be reliable. Furthermore, nutrient profiling systems such as the recently introduced Food Compass [48], which includes 54 attributes, can be very complex for general use; a balance in including additional components is crucial for any model with the ambition of being implemented into food policies.

Using the sale-weighting approach, we observed that the availability of foods in the supply does not necessary reflect the sales. Moreover, adjusting for market-share differences by sale-weighting notably affected the agreement between models. Sale-weighting deepened the differences, especially in food categories with lower agreement between models. This was most notable in Cooking oils, where sunflower oils and mixed vegetable oils have the largest market share, but grades for these oils differ notably between models (mostly D for NS, and 3.5–4 * for HSR). Sales data included in our study only covered the number of products sold in a year, which did not involve information on what impacted the sales and purchasing decisions of consumers. Normalizing such data based on brand marketing, price of a product, product packaging, taste, etc. would not only give us further insight into consumers decision but would also be valuable for FOP research. It would be especially beneficial for examining the use of FOP as a marketing tool and how different FOP schemes agree in this context.

The European Scientific Committee recently issued a report, which suggested further updates of the NS algorithm [49]. Based on this report, NS will change the algorithm for solid foods more similarly to HSR. The scales will be extended for most nutrients, which should enable NS to be more aligned with nutritional recommendations. The update also addresses some challenges exposed in this study, for example for cooking oils, cheese, and nuts and seeds, which will be able to get a higher grade. The report also announced further changes in the category of beverages, which are still under revision and should be published soon. Even though we observed that NS and HSR are mostly aligned for beverages, differences were observed in the discriminatory ability of flavoured waters, some juices, and beverages with non-caloric sweeteners. Recent studies based on a French NutriNet-Santé cohort showed that the intake of non-caloric sweeteners could be potentially associated with increased cardiovascular risk and increased cancer risk [50,51]. Together with the increasing consumption of beverages with non-caloric sweeteners [52] and unfavourable WHO opinion in the draft guidelines [53], changes in the NS algorithm could be expected for these beverages.

The main advantages of this study are the use of a large representative dataset of prepacked products (n = 17,226) and the performance of a comparison of NS and HSR across the whole food supply, which was not examined before. Furthermore, access to 12-month nation-wide sales data on the product level enabled us to also account huge market-share differences in the food supply.

The study limitations also need to be noted. Nutritional composition data needed for nutrient profiling were not always available for all parameters. Therefore, data such as dietary fibre content, % FVNL, and calcium content were estimated with the use of previously established methods. Furthermore, while sales data were available for the majority of foods, this was not the case for some foods—particularly for those marketed in discount stores. To avoid methodological mistakes, we compared the distribution of the whole sample with the dataset for which sales data were available, and no notable differences were found. Such an approach was also used previously [26]. We should also mention that we combined ten HSR grades into five to enable more meaningful comparison between NS and HSR. This was done based on the previous methodology and was also considered in the interpretation of the results. Finally, we should mention that NS and HSR were developed for two different markets, in different continents, with somewhat different dietary patterns, food supply, and public health priorities. The selection of a tested branded food dataset (in our case, this was a branded food dataset, compiled in Slovenia as an European Union country) therefore affected the study results. Since both models are gaining interest in other markets (HSR in India [54], and NS in Latin America [55]), further studies in other food environments would be useful.

## 5. Conclusions

This study highlighted the high discriminatory ability of two grading FOPNL schemes (NS and HSR) in most food (sub)categories. Both FOPNL models are highly compliant, with a few divergences in some subcategories. We observed that even though these two models do not always grade products equally high, the trend of ranking is mostly similar. However, the observed differences highlighted some challenges in the rating systems. Major differences were observed in categories Cooking oils and Cheese and processed cheese. Sales data showed that sales do not always follow the offer, which sometimes resulted in deepened differences between FOPNL schemes after accounting market-share data. International harmonization can support the further development of grading type nutrient profiling models for use in FOPNL, and make those acceptable for more stake-holders, which will be crucial for their successful regulatory implementation as part of efficient and useful food labelling.

This study also highlighted challenges in monitoring pre-packed products and examining the use of FOPNL schemes on real-life data. Missing and non-sufficient data limit comparison between different FOPNLs, which is especially evident in more complex nutrient profiling schemes.

## Figures and Tables

**Table 1:** Differences between Nutri-Score and Health Star Rating nutrient profiling models.

	Nutri-Score	Health Star Rating
Number of grades	5 (A “healthiest”–E “least healthy”)	10 (5 * “healthiest”–0.5 * “least healthy”) ^c^
Graphic representation	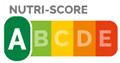	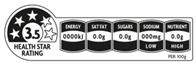
Profiling categories	1: Beverages 2: Foods 3: Added fats 4: Cheese	1: Non-dairy beverages 2: Foods 3: Oils and spreads 1D: Dairy beverages 2D: Dairy foods 3D: Cheese
Maximum points		
Energy	10	10 or 11 ^a^
Saturated fats	10	30
Total sugars	10	10 or 25 ^a^
Sodium	10	30
% FVNL ^b^	5 or 10 ^a^	8 or 10 ^a^
Proteins	5	15
Fibre	5	15
Specific profiling characteristics	Includes rapeseed, walnut, and olive oil as % FVNL ^b^ Automatically grades plain water: A (dark green) Minimum % FVNL ^b^ relevant for profiling is 40% Does not include concentrated juices as part of % FVNL ^b^ (except for 100% rehydrated juices)	Special conditions for profiling dairy products and imitates Minimum % FVNL relevant for profiling is 25% Automatically grades: ○ plain water: 5 * ○ unsweetened flavoured water: 4.5 * ○ fresh and minimally processed (canned and frozen) fruit and vegetables: 5 * Profiles water-based ice confections and jelly desserts as 1: Non-dairy beverages

^a^ depending on the profiling category. ^b^ % of fruit, vegetables, nuts, and legumes. ^c^ * refers to stars of Health Star Rating grades.

**Table 2:** Sample description of Slovenian branded food database (2020) used for nutrient profiling with Nutri-Score and Health Star Rating.

Category	Number of Products	% of the Sample
Total	17,226	100
Beverages	1822	10.6
Bread and bakery products	2304	13.4
Cereal and cereal products	547	3.2
Confectionery	2283	13.3
Convenience foods	750	4.4
Dairy and imitates	3203	18.6
Edible oils and emulsions	587	3.4
Fish and fish products	577	3.3
Fruit and vegetables	1452	8.4
Meat and meat products	1890	11.0
Sauces and spreads	1207	7.0
Snack foods	604	3.5

## Data Availability

The datasets presented in this article are not readily available because restrictions apply. The raw data supporting the conclusions of this article will be made available by the authors without undue reservation (without disclosure of specific brands or retailers).Sales data were obtained for internal use only and can be ordered directly from retailers. Requests to access the datasets should be directed to Igor Pravst (igor.pravst@nutris.org).

## Supplementary Materials

The following supporting information can be downloaded at: https://www.mdpi.com/article/10.3390/ijerph20053980/s1, Supplementary Material S1: Offering and sales-weighted distribution and alignment between Nutri-Score and Health Star Rating across 2020 Slovenian food supply.
