# Supplementary material for: Comparison of Nutri-Score and Health Star Rating Nutrient Profiling Models Using Large Branded Foods Composition Database and Sales Data
Source: Int J Environ Res Public Health. 2023 Feb 23;20(5):3980. doi: 10.3390/ijerph20053980 (PMC10002453; doi:10.3390/ijerph20053980)
Supplement: Supplementary file 1 [file ijerph-20-03980-s001.zip › ijerph-2106661-Supplementary.pdf]

Supplementary Material S1. Offering and sales-weighted distribution and alignment between Nutri-Score and Health Star Rating across 2020 Slovenian food supply.

	Nutri-Score [a] (%)					Health Star Rating [a] (%)							
**Category**	**A**	**B**	**C**	**D**	**E**	**4.5-5 ***	**3.5-4 ***	**2.5-3 ***	**1.5-2 ***	**0.5-1 ***	**% of Agreement [a]**	**κ**	**rho [b]**
**Total (n = 17,226)**	**10/26**	**12/26**	**20/8**	**32/18**	**25/21**	**12/31**	**21/27**	**16/7**	**22/11**	**29/24**	**70/81**	**0.62**	**0.87**
**Beverages (n = 1822)**	**10/43**	**11/5**	**17/5**	**22/11**	**39/37**	**12/43**	**10/4**	**18/6**	**23/11**	**38/36**	**94/98**	**0.91**	**0.98**
Juices (n = 349)	0/0	16/3	60/81	21/15	3/1	0/0	16/3	59/80	23/16	3/0	94/98	0.89	0.94
Nectars (n = 228)	0/0	4/2	8/0	9/7	79/91	0/0	4/3	13/4	14/15	70/78	86/81	0.57	0.85
Soft drinks (n = 1057)	0/0	13/10	8/5	29/20	50/66	3/1	11/9	8/5	29/20	49/65	94/98	0.91	0.99
Waters (n = 188)	100/100	0/0	0/0	0/0	0/0	100/100	0/0	0/0	0/0	0/0	100/100	/	/
**Bread and bakery products (n = 2304)**	**7/5**	**8/34**	**12/8**	**34/24**	**40/28**	**2/1**	**15/41**	**13/11**	**33/23**	**37/25**	**82/85**	**0.74**	**0.93**
Biscuits (n = 1129)	0/0	1/1	6/5	33/33	60/61	0/0	2/3	8/4	35/41	55/52	88/87	0.78	0.87
Bread (n = 247)	28/10	46/80	19/6	6/4	0/0	6/1	74/90	16/8	3/1	0/0	68/85	0.47	0.71
Cakes, muffins & pastry (n = 543)	1/0	1/1	8/3	49/50	42/46	0/0	3/1	11/10	47/45	39/44	85/90	0.75	0.84
Crispy bread (n = 291)	23/17	9/11	32/41	32/26	4/5	9/9	32/31	29/41	26/13	4/6	63/61	0.52	0.89
Other (n = 94)	10/10	24/32	19/21	40/34	6/2	1/1	34/42	21/34	38/22	5/1	82/75	0.75	0.95
**Cereal and cereal products (n = 547)**	**36/31**	**8/8**	**39/35**	**17/27**	**0/0**	**29/24**	**28/20**	**33/36**	**10/20**	**0/0**	**69/79**	**0.57**	**0.86**
Breakfast cereals (n = 484)	28/18	9/9	44/41	19/32	0/0	20/11	31/24	38/42	11/23	0/0	66/75	0.53	0.83
Cereal flakes and bran (n = 63)	97/100	2/0	2/0	0/0	0/0	94/99	5/1	2/0	0/0	0/0	94/99	0.31	0.35
**Confectionery (n = 2283)**	**1/0**	**2/1**	**7/1**	**34/36**	**56/62**	**1/0**	**3/1**	**7/1**	**19/9**	**69/89**	**80/72**	**0.63**	**0.76**
Chocolate and sweets (n = 2104)	1/0	2/1	7/1	29/32	61/67	1/0	4/1	7/1	14/4	74/95	79/71	0.59	0.74
Jelly candy (n = 179)	1/0	1/0	4/0	93/100	0/0	1/0	1/0	6/1	79/86	13/13	86/87	0.46	0.61
**Convenience foods (n = 750)**	**20/40**	**27/25**	**34/24**	**18/11**	**2/0**	**2/0**	**51/68**	**34/23**	**11/8**	**2/0**	**68/53**	**0.56**	**0.87**
Pizza (n = 73)	0/0	16/15	53/67	30/18	0/0	0/0	22/24	62/66	16/9	0/0	81/82	0.67	0.81
Pre-prepared salads and sandwiches (n = 137)	17/5	25/28	35/39	23/27	0/0	0/0	47/35	38/41	15/23	0/0	69/90	0.56	0.88
Ready meals (n = 304)	19/31	34/46	30/19	16/4	1/0	2/0	59/81	29/16	7/2	3/0	65/62	0.50	0.83
Side dishes (n = 236)	29/61	22/5	33/21	14/12	3/0	4/0	50/69	30/22	12/9	3/0	67/32	0.57	0.89
**Dairy and imitates (n = 3203)**	**11/16**	**24/64**	**20/7**	**38/13**	**6/1**	**27/33**	**29/52**	**14/5**	**12/2**	**18/8**	**53/70**	**0.42**	**0.64**
Cheese and processed cheese (n = 827)	0/0	1/0	12/10	82/87	5/3	35/44	28/22	8/9	6/4	22/21	8/5	0.01	0.38
Cheese imitates (n = 34)	0/0	3/0	3/0	12/30	82/70	0/0	3/0	3/0	6/0	88/99	94/70	0.78	0.82
Cottage cheese (n = 78)	47/26	33/62	19/12	0/0	0/0	73/80	22/20	5/0	0/0	0/0	60/35	0.30	0.75
Cream (n = 155)	0/0	0/0	11/24	86/76	3/0	0/0	0/0	11/7	8/2	81/91	14/03	0.03	0.26
Cream imitates (n = 38)	0/0	37/9	11/0	53/90	0/0	0/0	37/9	18/13	16/5	29/73	55/14	0.41	0.85
Desserts (n = 192)	9/6	21/22	53/59	16/13	2/0	9/6	35/61	36/19	6/3	14/11	72/49	0.61	0.88

Flavoured yogurt (n = 572)	8/3	46/80	45/17	1/0	0/0	19/33	54/56	24/11	2/0	0/0	69/64	0.50	0.76
Ice cream and edible ices (n = 568)	2/0	2/1	18/22	59/69	19/8	1/0	2/1	17/37	48/46	32/15	77/71	0.62	0.50
Milk & milk drinks (n = 237)	35/20	59/79	5/0	0/0	1/0	43/27	48/72	3/0	4/0	1/0	82/93	0.68	0.81
Milk imitates (n = 185)	18/12	71/86	3/1	7/1	1/0	16/9	74/90	2/0	8/1	1/0	94/96	0.85	0.92
Plain yogurt (n = 274)	38/25	50/67	11/8	0/0	0/0	83/90	8/2	9/7	0/0	0/0	53/33	0.26	0.61
Yogurt imitates (n = 43)	37/51	30/29	28/19	5/1	0/0	49/59	23/23	14/0	9/18	5/1	70/72	0.57	0.90
**Edible oils and emulsions (n = 587)**	**0/0**	**0/0**	**43/20**	**39/65**	**18/15**	**3/2**	**45/72**	**27/6**	**3/3**	**22/17**	**35/22**	**0.22**	**0.63**
Butter (n = 67)	0/0	0/0	0/0	9/3	91/97	0/0	0/0	0/0	4/1	96/99	93/98	0.41	0.44
Cooking oils (n = 465)	0/0	0/0	51/21	40/77	9/1	2/0	54/91	33/8	1/0	10/1	27/06	0.11	0.40
Margarine (n = 55)	0/0	0/0	27/36	67/63	5/1	15/19	22/28	9/2	18/32	36/18	27/35	0.13	0.72
**Fish and fish products (n = 577)**	**16/7**	**21/25**	**25/19**	**36/49**	**2/1**	**7/3**	**50/47**	**17/22**	**18/28**	**8/1**	**55/58**	**0.41**	**0.81**
Canned fish and seafood (n = 387)	3/1	19/22	30/21	45/57	3/1	2/0	47/43	21/24	22/32	9/1	51/57	0.34	0.77
Processed chilled fish products (n = 81)	14/36	27/45	23/13	36/6	0/0	0/0	44/84	20/10	26/3	10/3	73/58	0.63	0.94
Unprocessed chilled fish (n = 109)	64/57	23/36	9/0	4/7	0/0	29/42	66/51	4/7	1/0	0/0	54/78	0.30	0.55
**Fruit and vegetables (n = 1452)**	**28/41**	**20/26**	**37/22**	**15/10**	**0/0**	**34/46**	**29/30**	**18/11**	**17/11**	**2/1**	**67/81**	**0.57**	**0.84**
Canned fruit (n = 115)	23/12	65/87	10/1	2/1	0/0	7/9	78/85	11/5	3/1	0/0	78/93	0.53	0.71
Dried fruit (n = 279)	9/14	23/29	57/51	11/6	0/0	11/14	33/38	31/33	16/8	8/7	59/78	0.43	0.79
Dried vegetables (n = 21)	67/11	19/5	14/84	0/0	0/0	81/16	19/84	0/0	0/0	0/0	71/11	0.33	0.78
Frozen fruit (n = 43)	100/100	0/0	0/0	0/0	0/0	100/100	0/0	0/0	0/0	0/0	100/100	/	/
Frozen vegetables (n = 129)	100/100	0/0	0/0	0/0	0/0	100/100	0/0	0/0	0/0	0/0	100/100	/	/
Jam and spreads (n = 324)	3/1	6/2	51/38	40/59	0/0	0/0	10/2	37/26	52/71	0/0	82/86	0.70	0.82
Nuts and fruit mixes (n = 109)	13/1	41/60	41/40	5/0	0/0	28/16	58/84	12/0	3/0	0/0	48/46	0.23	0.65
Nuts and seeds (n = 432)	33/29	19/32	37/33	11/5	1/1	53/53	32/40	8/4	6/1	2/1	50/49	0.31	0.78
**Meat and meat products (n = 1890)**	**7/5**	**4/4**	**8/11**	**37/58**	**44/23**	**4/3**	**15/15**	**6/8**	**33/58**	**42/17**	**71/73**	**0.57**	**0.85**
Animal fat products (n = 69)	0/0	0/0	0/0	0/0	100/100	0/0	1/0	3/0	14/3	81/97	81/97	/	/
Meat alternatives (n = 158)	37/55	15/17	24/18	21/11	3/0	16/24	56/59	12/7	13/9	3/0	51/42	0.39	0.77
Processed meat & meat spreads (n = 1584)	1/1	2/4	7/11	42/63	48/22	0/0	10/13	6/8	38/63	46/15	72/73	0.55	0.79
Unprocessed meat (n = 79)	71/89	20/0	9/11	0/0	0/0	53/71	47/29	0/0	0/0	0/0	73/71	0.50	0.67
**Sauces and spreads (n = 1207)**	**10/10**	**11/11**	**33/23**	**30/20**	**15/35**	**5/4**	**21/21**	**27/21**	**25/25**	**22/29**	**71/76**	**0.62**	**0.83**
Mayonnaise and dressings (n = 108)	0/0	0/0	14/3	41/7	45/89	0/0	0/0	15/3	63/55	22/42	72/53	0.55	0.73
Nut spreads (n = 68)	28/41	3/3	37/44	26/12	6/0	37/46	28/42	7/5	24/8	4/0	57/49	0.47	0.94
Sauces (n = 785)	10/15	14/19	38/33	30/31	8/1	4/6	22/31	30/33	23/27	21/3	71/78	0.61	0.81
Sweet spreads (n = 95)	0/0	0/0	3/0	22/5	75/95	0/0	2/0	5/1	16/1	77/98	89/96	0.73	0.84
Vegetable spreads (n = 151)	13/12	19/13	38/68	29/8	0/0	1/0	38/50	42/45	16/5	4/0	67/60	0.54	0.87
**Snack foods (n = 604)**	**2/2**	**2/0**	**21/24**	**53/51**	**21/23**	**1/2**	**6/1**	**27/32**	**45/56**	**20/9**	**75/68**	**0.62**	**0.82**

κ—Cohen's Kappa; rho—Spearman Rank Correlation; [a]—presented as availability of products/sales-weighted; [b]—all results are statistically significant ($p < 0.05$). * refers to stars of Health Star Rating grades.
